# Hyperbaric Oxygen Therapy of an Adolescent Stem Cell Transplantation Recipient with Hemorrhagic Cystitis and BK Virus

**DOI:** 10.1155/2020/3465412

**Published:** 2020-05-13

**Authors:** David López, Abdullah Alismail, Laren D. Tan

**Affiliations:** ^1^Department of Cardiopulmonary Sciences, School of Allied Health Professions, Loma Linda University, California, USA; ^2^Department of Medicine, School of Medicine, Loma Linda University, Loma Linda, USA; ^3^Loma Linda University Health, Division of Pulmonary, Critical Care, Hyperbaric, Allergy and Sleep Medicine, USA

## Abstract

Hyperbaric oxygen therapy (HBOT) continues to show effectiveness in the treatment of several diseases and benefits such as fibroblast proliferation, capillary angiogenesis, and decreasing edema, especially in hemorrhagic cystitis (HC). We report a case of a 15-year-old male with chronic myelogenous leukemia status posthaploidentical stem cell transport with BK virus in the United States to be treated by HBOT. The patient received a total of 30 HBOT treatments for 90 minutes at 2 ATA. After HBOT treatments, patient showed signs of improvements such as cessation of hemorrhage cystitis. The findings of this case support and shows that there is growing evidence for the use of HBOT as adjunctive therapy for patients with BK virus associated with HC after stem cell transplantation.

## 1. Introduction/Background

The current indications and uses of medical hyperbaric oxygen are continuously being studied and investigated as an adjunctive care application associated with the various clinical and physiologic benefits of hyperbaric oxygen therapy (HBOT). As defined by the Undersea and Hyperbaric Medical Society (UHMS), (HBOT) is a clinical intervention where the patient is in a hyperbaric chamber where the pressure must equal or exceed 1.4 ATA (Atmosphere Absolute) while breathing near 100% oxygen [1]. HBOT can be carried out in either a monochamber (single person) or a multiplace chamber (more than 2 patients at a time). Generally, HBOT treatments consist of the patient breathing 100% oxygen ranging from 1.5 to 2 hours at times alternating oxygen breaks depending on the indication and prescribed use.

As of 2011, the UHMS has 14 approved indications for HBOT therapy with the most common clinical indications being for the enhancement of difficult healing wounds [1–5], necrotizing soft tissue infections (gas gangrene and necrotizing fasciitis), and decompression sickness [5, 6]. The most recent approved use of HBOT therapy is for the treatment of idiopathic sudden sensorineural hearing loss [7] approved by the UHMS in 2014 [5, 8]. To date, there are relatively few reported contraindications and only mild side effects associated with the pressurized chambers and high oxygen content of HBOT. There are other contraindications, some related to chemotherapy such as bleomycin [9].

The most common indications and effective treatments of HBOT are in the areas of damaged and injured tissues from various causes, for example, but not limited to, chronic illnesses such as burns, cell damage from diabetes, radiation injury, tissue hypoxia, enhancing tissue graft (to include bone) effectiveness, and generally poor healing conditions [10–12].

The elevated levels of available oxygen and partial pressure of arterial oxygen provide the main benefits of HBOT in clinical practice that addresses these areas of inadequate or poor tissue healing. The pharmacological and physiologic effects of HBOT have direct and indirect mechanisms and effects on reactive oxygen species (ROS) most beneficial to that of wound healing and antibacterial treatments [8, 13]. Of these mechanisms and effects, those of fibroblast proliferation via fibroblast growth factor (FGF), capillary angiogenesis, decreasing edema, the upregulation of vascular endothelial growth factor (VEGF), decreasing nitric oxide (NO) leading to vasoconstriction, cell proliferation and migration, wound granulation, and improved epithelial production have shown promise with HBOT [8].

Among the conditions that are due to tissue damage and poor healing is hemorrhagic cystitis (HC). Although typically associated with radiation damage, HC is also considered to be one of the potential adverse effects related to stem cell transplantation (SCT). It has been reported that the incidence of HC can be as high as 50% or more of SCT procedures and represents a noteworthy morbidity risk to these patients [14]. Further, the effects of HC on the bladder wall consist of not only inflammation, bleeding, irritation, and pain but also infections, scarring, stiffening of the bladder, and decreased volume capacity. This added complication to SCT, among other conditions, increases the potential morbidity and mortality of the patient, which is especially problematic in the pediatric population [11, 15–19].

Moreover, BK virus (BKV) commonly presents as a polyomavirus latent opportunistic infection in SCT patients, a major contributing factor to the onset of HC of up to 50% or more in this population [14, 20]. It is also estimated that the BK virus is present in up to 80% of the general population, expressed as opportunistic in the immunosuppressed and immunocompromised patients [14, 20]. Late-onset HC is also attributed to BKV in this population of immunosuppressed, immunocompromised individuals [14, 20]. Therefore, based on recent and initial findings regarding the use of HBOT in patients undergoing SCT and their concomitant posttransplant morbidities, a consult was requested resulting in this nonusual adjunctive treatment approach [18]. To the authors' knowledge, the uniqueness of this case report is among one of the three reported and treated cases in the United States of America [21].

## 2. Case

This is a case of a 15-year-old male with chronic myelogenous leukemia (CML) status posthaploidentical stem cell transplant (HSCT). In addition, the patient had an infectious disease history of strep viridans, fungal prophylaxis, CMV viremia, and adenovirus. BK viremia was initially asymptomatic with the patient developing hemorrhagic cystitis soon after the procedure. The patient was started on a standard treatment of cidofovir 1 mg/kg/dose with probenecid and leflunomide (loading dose of 100 mg daily × 3 days, then 40 mg daily). In addition, the patient was also placed on levofloxacin for synergistic treatment. The platelet count on admission was 49 bil/L, red blood cell count 3.06 tril/L hemoglobin 9.8 g/dl, hematocrit 28%, and RDW 23.2%. Also, the BK virus was greater than 2 million BK on admission. The admission diagnosis was poor medication compliance-induced HC due to BK in an immunocompromised state associated with SCT, hypomagnesemia, and pancytopenia.

Despite the standard of care therapy for HC after stem cell transplantation (cidofovir, leflunomide, and levofloxacin, continuous bladder irrigation), the patient's HC continued to worsen: increasing BK virus flow counts, hematuria, and no improvement of RBC (2.70 tril/L), Hgb (8.1 g/dL), Hct (22.4%), and RDW (15.1%). HBOT was available and prescribed to the patient. On day 1, the initial order of HBOT, using a monochamber, was 2 ATA for 90 minutes per treatment with an assessment and evaluation of the patient's condition at treatment 10. Treatment was prescribed daily Monday through Friday. At day 10, treatment 10, the patient was evaluated indicating the treatment was tolerated. In addition, some improvement in the hematuria from the nephrostomy tubes was noted (BK virus count 19460 copies/mL down from 2 million); therefore, additional 10 treatments were prescribed to the patient. On day 20, following treatment 20, an evaluation shows the BK virus count trending down to 2362 copies/mL with improvement in the hematuria. Therefore, additional 10 treatments were added to support the continuing down trend of the BK virus count and decreasing hematuria, along with the patient showing significant overall improvement. At day 30, HBOT was discontinued after treatment 30 based on the resolution of HC signs and symptoms (hematuria and normal or absent BK virus count).

## 3. Discussion and Conclusion

Hemorrhagic cystitis is well-reported and documented in the literature as one of the frequent complications of hematopoietic SCT, and it is the result of diffuse inflammation and vascular injury of the bladder mucosa due to immunosuppression and opportunistic infections [16, 18]. Late-onset HC is a dangerous and often life-threatening condition that it is frequently associated with reactivation of various viruses such as adenovirus, polyoma, and BKV [14, 22]. To further assist in healing, HBOT can be used to treat damaged tissue from hypoxic injury and wound healing through stimulation of angiogenesis and fibroblast proliferation primarily in the HSCT-BKV-HC patient [14, 22–26].

The findings of this case support similar reported cases on the use of HBOT in the treatment of patients with BKV associated with SCT [21, 23, 25]. Reported cases have shown high cure rates [20]. Savva-Bordalo et al. reported similar findings with clinical resolution of hematuria in 94% of the 16 patients they treated with HBOT [20]. To date, there continues to be growing evidence of HBOT being an adjunctive therapy for patients with BK virus-associated HC after SCT [14, 20, 21, 27]. Although the primary reason for using HBOT, in this case, was due to persistent HC despite standard of care therapy [20], it is also the third reported case in the United States, to the authors' knowledge, and provides added support as an option for HBOT in these cases. Kaur et al. [21] reported having one of the two cases, a 17 year old, with 20 HBOT treatments, where 30 treatments were delievered in this case. This case adds to the current evidence of using HBOT for BKV-associated HC post-SCT from different countries around the world. Although prospective, randomized controlled trials are needed to establish a better understanding of this therapy, healthcare providers are encouraged to consider HBOT as a possible therapy in these cases due to the added evidence of several case studies and reports worldwide [8, 20, 21, 25, 26, 28–31].
